# Fully Guided Implant Placement for Rehabilitation of Congenital Maxillary Lateral Incisor Agenesis Using Cone-Beam Computed Tomography (CBCT) and Intraoral Digital Scanning: A Case Report

**DOI:** 10.7759/cureus.113581

**Published:** 2026-07-29

**Authors:** Keily J Portillo Avila, Maribel D Lopez Medal, Yelitza Rojas, Sandra K Estupinan Paredes, Ana M Restrepo Roldan

**Affiliations:** 1 Periodontics, Dental Loft, San Pedro Sula, HND; 2 Dentistry, Miami Modern Dental Clinic, Miami, USA; 3 Dentistry, Family Dental Home, Casallberry, USA; 4 Dentistry, Centro Medico Cafam, Bogotá, COL; 5 Dentistry, Aeticodontologia, Medellin, COL

**Keywords:** computer-aided design/computer-aided manufacturing (cad/cam), cone beam computed tomography (cbct), dental implant, digital workflow, guided implant surgery, hypodontia, intraoral scanner, maxillary lateral incisor, surgical guide, tooth agenesis

## Abstract

Computer-guided implant surgery has significantly improved the accuracy and predictability of implant placement through the integration of cone-beam computed tomography (CBCT), intraoral digital scanning, and computer-aided design/computer-aided manufacturing (CAD/CAM) technology. This case report describes the fully guided digital workflow for the rehabilitation of congenital hypodontia of the maxillary right lateral incisor in a 25-year-old healthy male. Clinical examination, panoramic radiography, CBCT imaging, and intraoral digital scanning were performed to evaluate the edentulous site and facilitate prosthetically driven virtual implant planning. A tooth-supported three-dimensional (3D) printed surgical guide was digitally designed and fabricated to accurately transfer the virtual treatment plan to the clinical setting. Guided osteotomy and implant placement were completed according to the digital treatment plan, achieving excellent primary stability and precise 3D implant positioning. Following a six-month osseointegration period, the implant was restored with a definitive implant-supported crown. Clinical and radiographic evaluations confirmed successful osseointegration, accurate implant positioning, uneventful healing, and favorable esthetic and functional outcomes. This case demonstrates that the integration of CBCT, intraoral digital scanning, virtual implant planning, and CAD/CAM surgical guide fabrication provides a predictable, minimally invasive, and prosthetically driven approach for implant rehabilitation, improving surgical precision, treatment safety, and restorative outcomes.

## Introduction

Dental implants have become the preferred treatment for replacing missing teeth because they provide predictable long-term functional and esthetic outcomes while preserving adjacent teeth and supporting alveolar bone. The success of implant therapy depends largely on accurate three-dimensional (3D) implant positioning, which influences osseointegration, prosthetic restoration, peri-implant tissue stability, and long-term maintenance [1-7].

Traditionally, implant placement has been performed using freehand surgical techniques based on two-dimensional radiographic assessment and clinical experience. Although successful in many cases, freehand surgery may result in deviations in implant angulation, depth, and position, potentially compromising prosthetic outcomes or increasing the risk of injury to adjacent anatomical structures. Advances in digital dentistry have significantly improved implant planning and surgical accuracy through the integration of cone-beam computed tomography (CBCT), intraoral digital scanning, computer-assisted implant planning, and computer-aided design/computer-aided manufacturing (CAD/CAM) technology [1-8].

The integration of CBCT imaging with intraoral digital scanning enables prosthetically driven virtual implant planning by combining 3D radiographic and surface data. This digital workflow facilitates precise evaluation of bone anatomy, implant position, angulation, restorative space, and emergence profile before fabrication of a patient-specific CAD/CAM surgical guide. The resulting surgical guide accurately transfers the virtual treatment plan to the clinical setting, allowing more precise, minimally invasive, and predictable implant placement [9-14].

Congenital absence (hypodontia) of the maxillary lateral incisor is one of the most common developmental dental anomalies and frequently presents both esthetic and functional challenges. Implant-supported rehabilitation after completion of craniofacial growth represents a conservative treatment option that restores function and esthetics without preparation of adjacent teeth [15-20].

This case report describes the complete digital workflow for rehabilitation of a congenitally missing maxillary right lateral incisor using CBCT-based virtual planning, intraoral digital scanning, CAD/CAM surgical guide fabrication, and fully guided implant placement, demonstrating the clinical application of digital technology to achieve accurate, predictable, and prosthetically driven implant rehabilitation.

## Case presentation

A 25-year-old healthy male (American Society of Anesthesiologists Physical Status Classification (ASA) I) presented with concerns regarding the congenital absence (hypodontia) of the maxillary right lateral incisor (tooth #7) and requested a fixed esthetic replacement. The patient reported no significant medical history, systemic diseases, medication use, smoking history, or contraindications to implant surgery. Clinical examination confirmed the congenital absence of tooth #7, with healthy periodontal tissues and adjacent teeth. Adequate mesiodistal space, favorable occlusion, and sufficient restorative space were present, making the patient an appropriate candidate for implant-supported rehabilitation.

**Figure 1:**
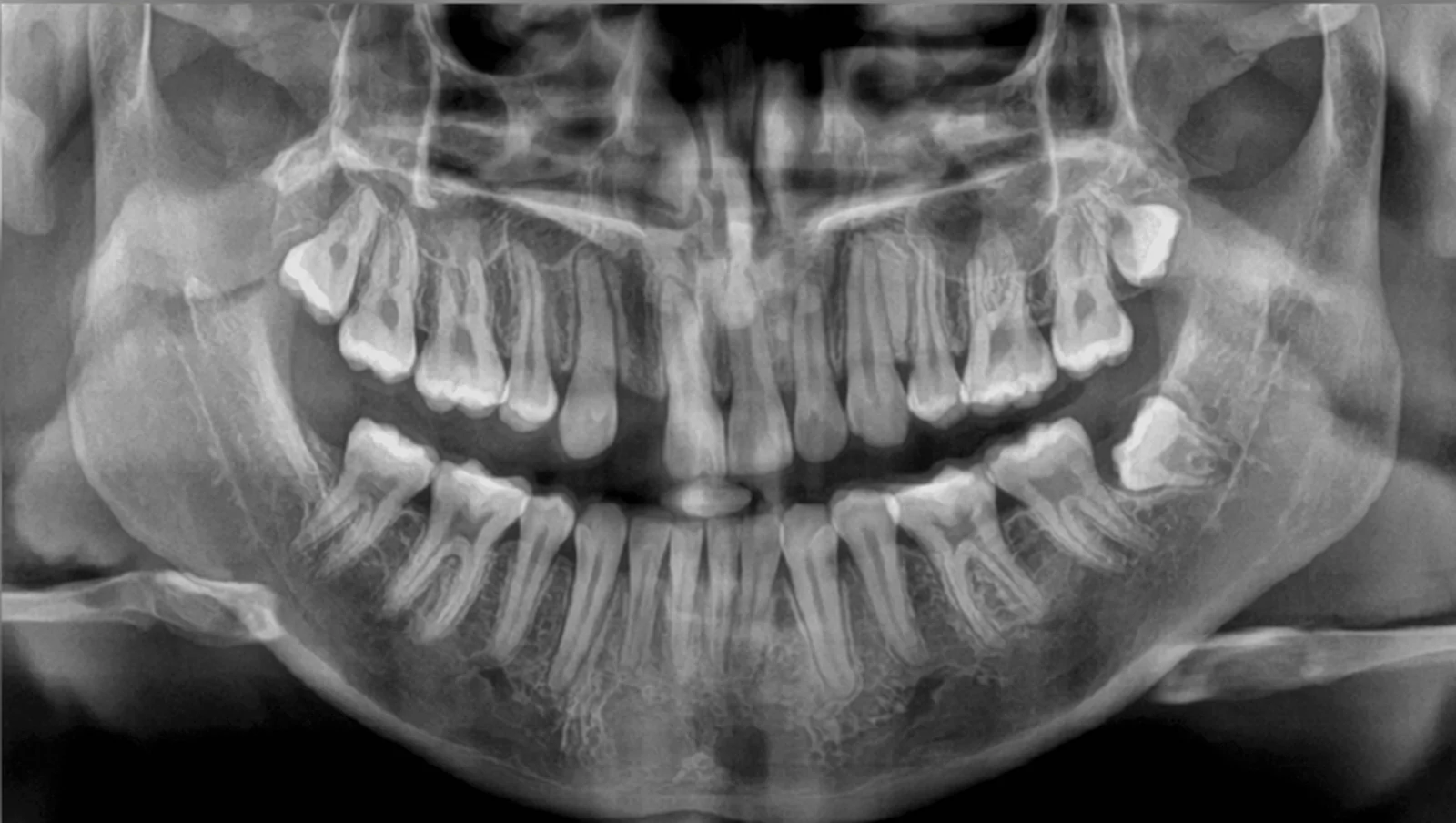
Preoperative panoramic radiograph demonstrating congenital hypodontia (agenesis) of the maxillary right lateral incisor (tooth #7). The edentulous site is evident between the maxillary right central incisor and canine. The remaining permanent dentition demonstrates normal development without radiographic evidence of associated pathology. This radiographic examination was used for the initial diagnostic assessment and prosthetically driven treatment planning prior to fully guided implant rehabilitation following completion of craniofacial growth.

**Figure 2:**
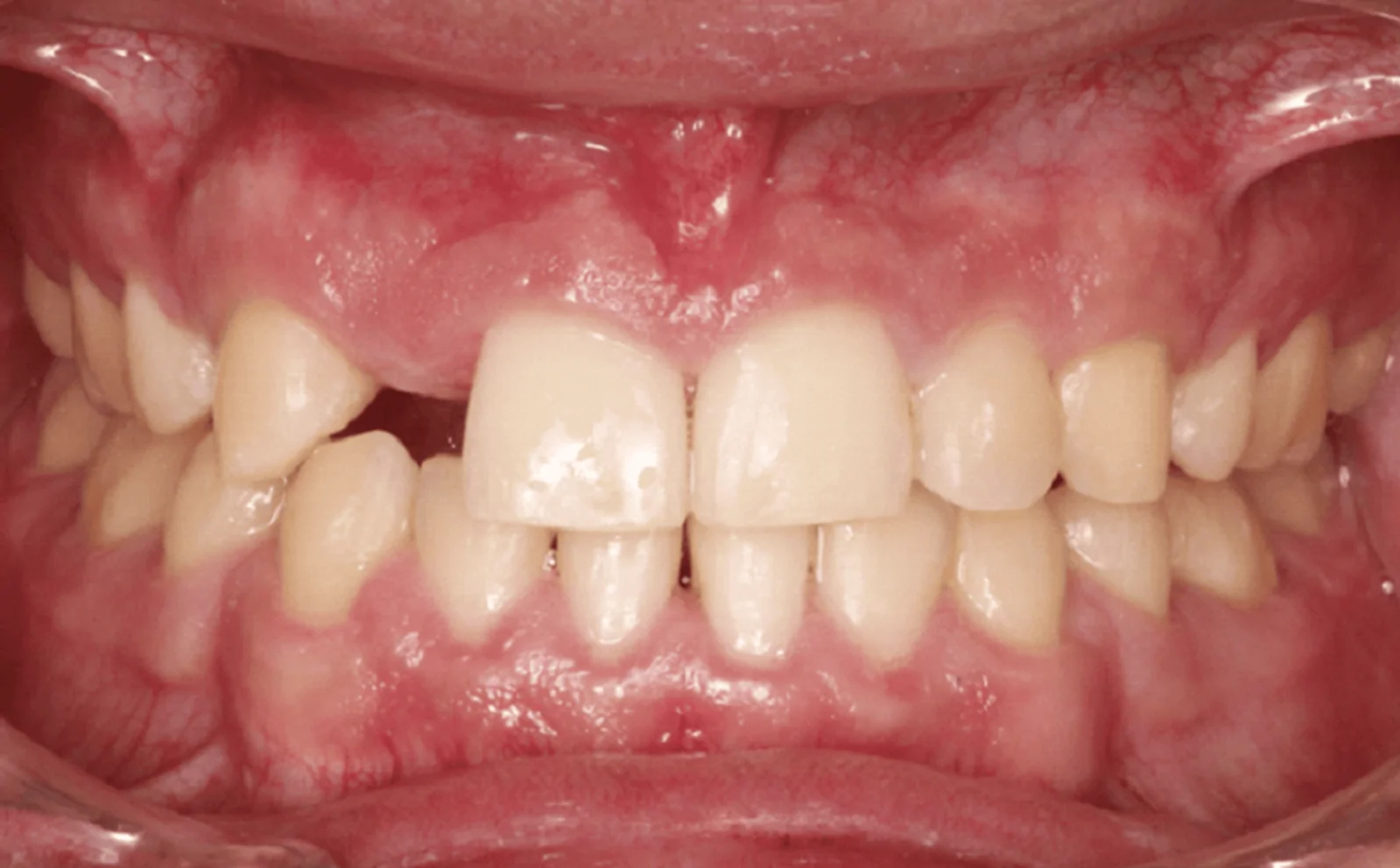
Preoperative frontal intraoral photograph demonstrating congenital hypodontia (agenesis) of the maxillary right lateral incisor (tooth #7). The edentulous space between the maxillary right central incisor and canine is clearly visible, with healthy surrounding soft tissues and adequate mesiodistal restorative space for implant-supported rehabilitation. This clinical examination served as the basis for prosthetically driven digital implant planning and fabrication of a computer-aided design/computer-aided manufacturing surgical guide.

A panoramic radiograph was obtained as part of the initial diagnostic evaluation and confirmed the congenital absence of the maxillary right lateral incisor without evidence of associated pathology involving the adjacent dentition or supporting bone (Figure 1). Clinical intraoral photographs demonstrated the edentulous space between the maxillary right central incisor and canine, with healthy soft tissues and adequate space for prosthetically driven implant placement (Figure 2).

**Figure 3:**
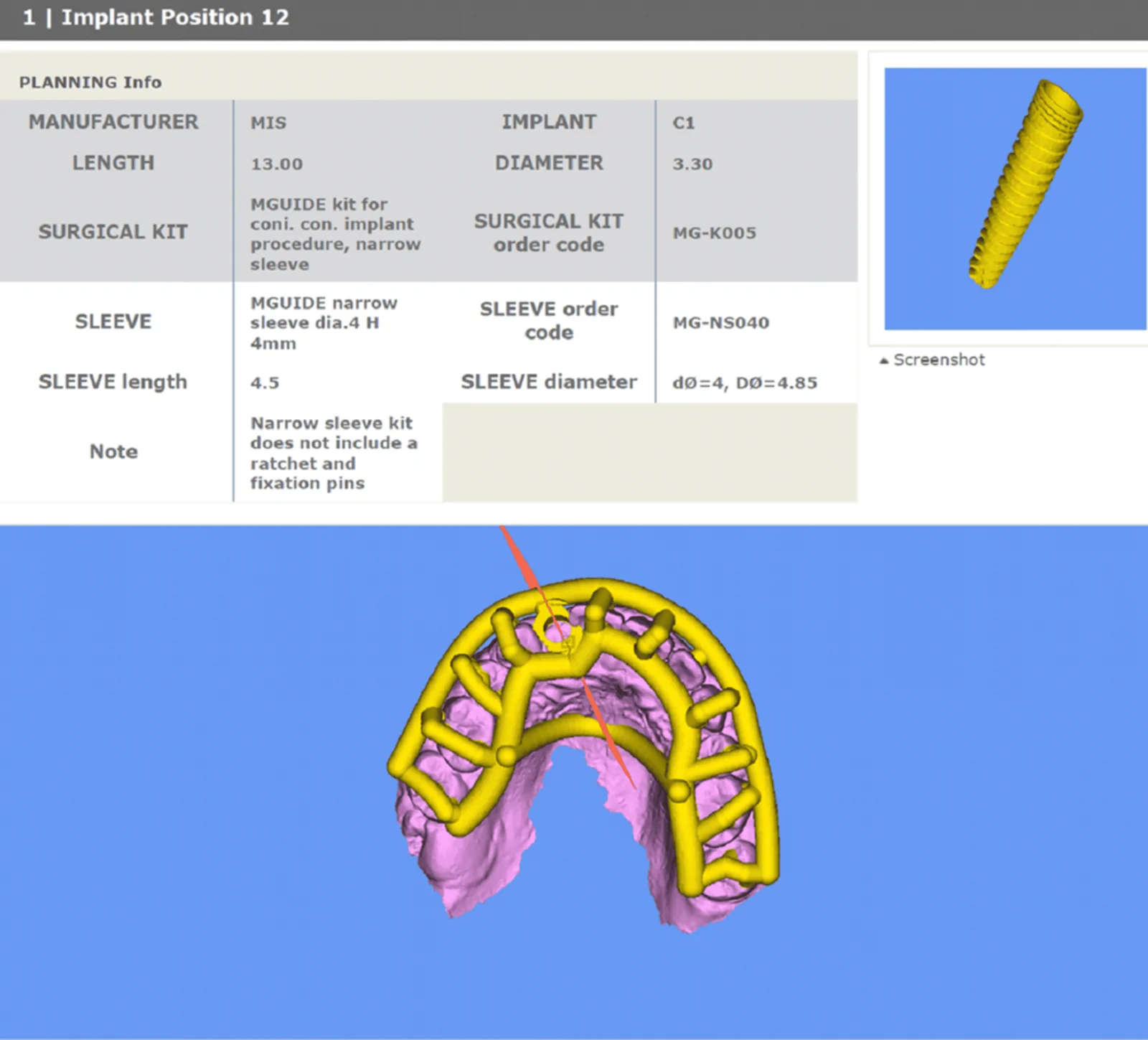
Prosthetically driven virtual implant planning and computer-aided design/computer-aided manufacturing (CAD/CAM) surgical guide design. Cone-beam computed tomography imaging and an intraoral digital scan were integrated using implant planning software to perform prosthetically driven three-dimensional (3D) virtual implant planning. The digital workflow enabled evaluation of the available bone volume, implant angulation, depth, restorative emergence profile, and relationship to the adjacent teeth before surgery. Based on the virtual treatment plan, a tooth-supported CAD/CAM surgical guide was designed for 3D printing to accurately transfer the planned implant position to the clinical procedure. The planned implant measured 3.3 mm in diameter and 13 mm in length.

A fully digital workflow was selected to optimize surgical accuracy and restorative planning. CBCT was performed to evaluate alveolar bone height, width, and morphology, as well as the relationship of the proposed implant site to adjacent anatomical structures. An intraoral digital scan was obtained to capture the maxillary and mandibular dentition and occlusal relationship. The CBCT data and intraoral digital scan were integrated using implant planning software to perform prosthetically driven 3D virtual implant planning. This digital workflow enabled precise evaluation of bone anatomy, implant position, angulation, depth, and restorative emergence profile before fabrication of the surgical guide (Figure 3).

**Figure 4:**
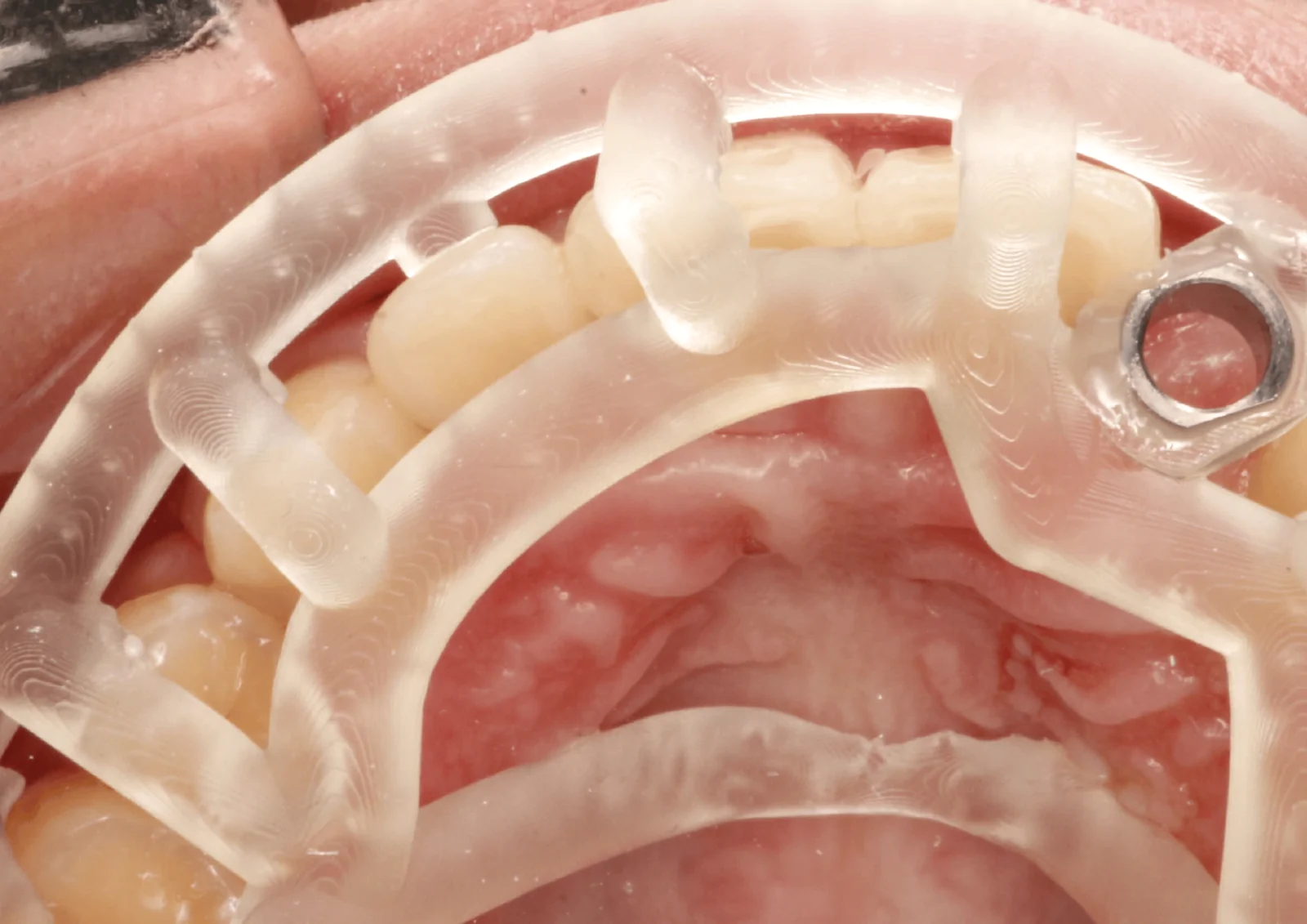
Intraoperative clinical photograph demonstrating the tooth-supported three-dimensional printed computer-aided design/computer-aided manufacturing surgical guide seated on the maxillary dentition before guided implant surgery. The guide was clinically verified for complete seating, passive fit, stability, and accurate adaptation to the adjacent teeth. The incorporated metallic guide sleeve was positioned over the planned implant site to direct the sequential osteotomy according to the prosthetically driven virtual treatment plan, ensuring precise transfer of the digital planning to the clinical procedure.

Based on the virtual treatment plan, a tooth-supported CAD/CAM surgical guide incorporating a metallic guide sleeve was designed and fabricated using 3D printing technology. The guide was clinically verified before surgery to ensure complete seating, stability, and accurate adaptation to the remaining dentition (Figure 4). 

**Figure 5:**
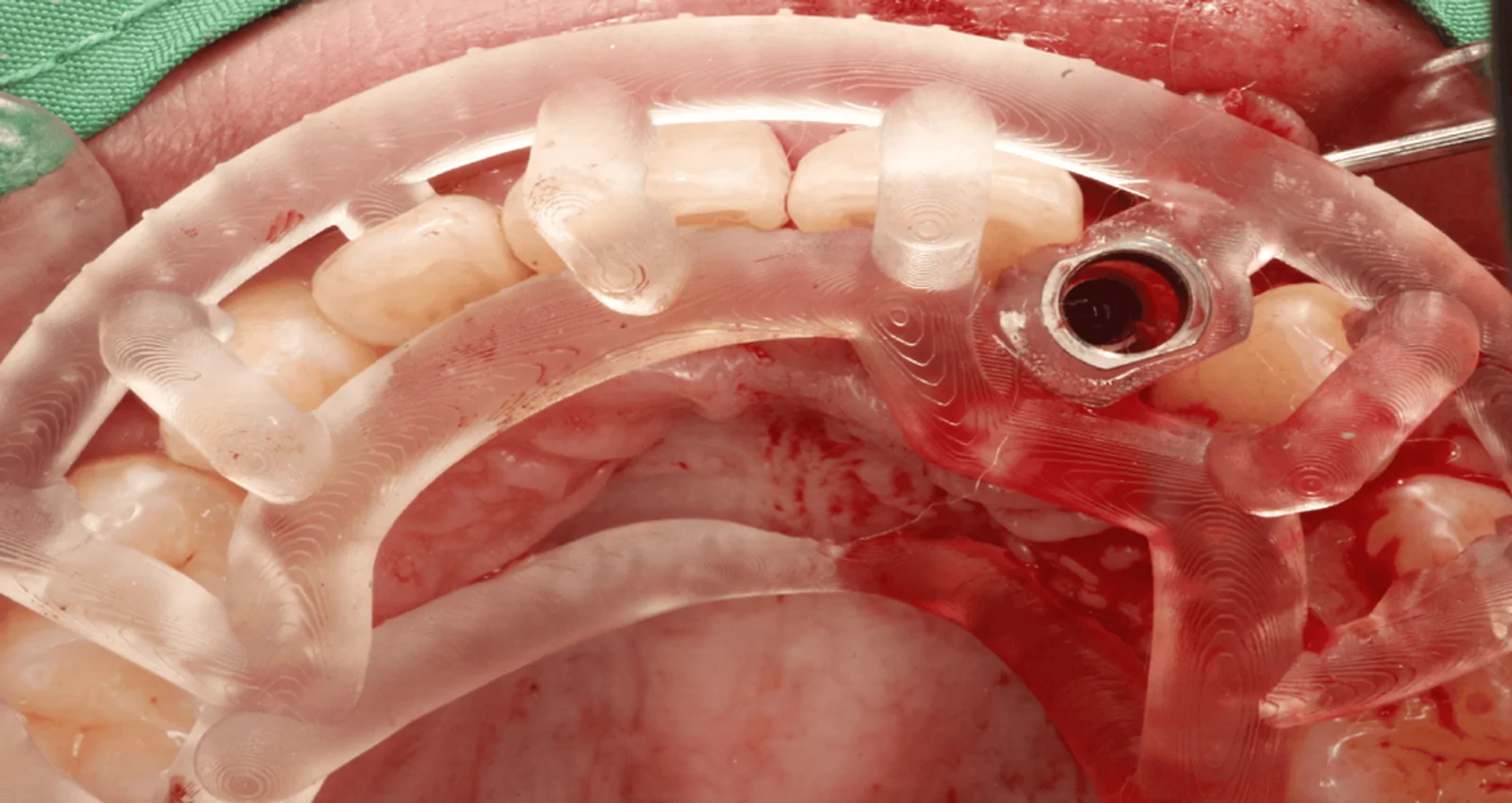
Intraoperative clinical photograph demonstrating guided osteotomy preparation using a tooth-supported three-dimensional (3D) printed computer-aided design/computer-aided manufacturing surgical guide. The metallic guide sleeve directed the sequential drilling protocol according to the prosthetically driven virtual implant plan, ensuring accurate 3D implant positioning while minimizing surgical deviation and preserving the surrounding anatomical structures. This fully guided approach facilitated precise transfer of the digital treatment plan to the clinical procedure prior to placement of the 3.3 × 13 mm dental implant.

Implant surgery was performed under local anesthesia using a fully guided surgical protocol. Following flap elevation, the surgical guide was positioned and its stability confirmed. Sequential osteotomy preparation was completed through the guide sleeves according to the manufacturer's guided drilling sequence. The implant was inserted following the virtual treatment plan, achieving excellent primary stability and precise 3D positioning. A healing abutment was connected, and the surgical site was irrigated and closed with interrupted sutures. No intraoperative complications occurred during the procedure (Figure 5). 

**Figure 6:**
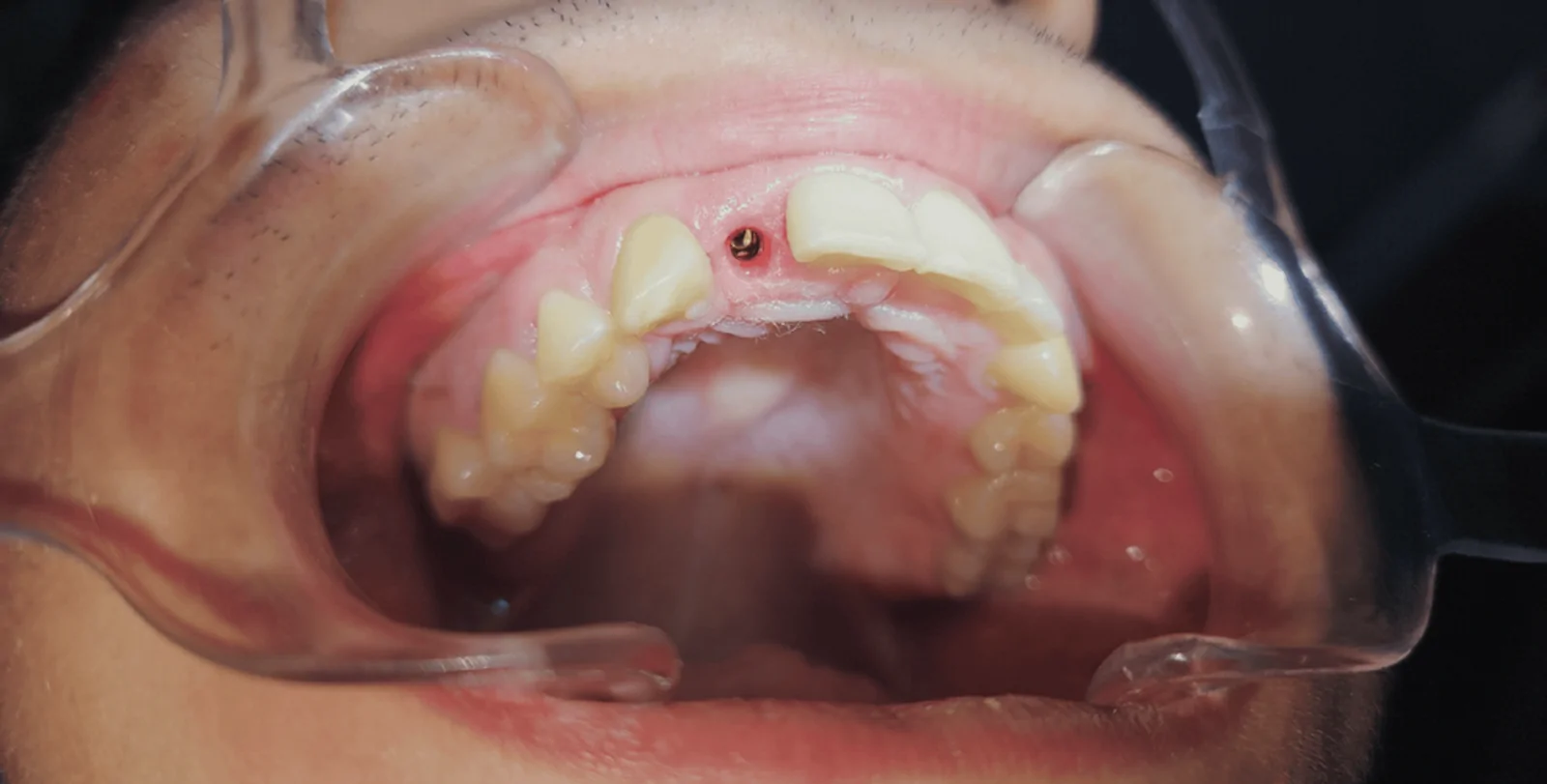
Intraoral clinical photograph obtained after a six-month osseointegration period before definitive prosthetic restoration, demonstrating successful integration of the dental implant placed in the site of the congenitally missing maxillary right lateral incisor (tooth #7). The healing abutment is visible emerging through healthy peri-implant soft tissues, exhibiting favorable soft tissue maturation and excellent clinical stability. These findings confirmed successful osseointegration before placement of the definitive implant supported crown.

The postoperative healing period was uneventful. Clinical follow-up demonstrated satisfactory healing of the peri-implant soft tissues without signs of infection, inflammation, wound dehiscence, or other complications. The healing abutment exhibited an appropriate transmucosal emergence profile with healthy peri-implant soft tissue adaptation (Figure 6). 

**Figure 7:**
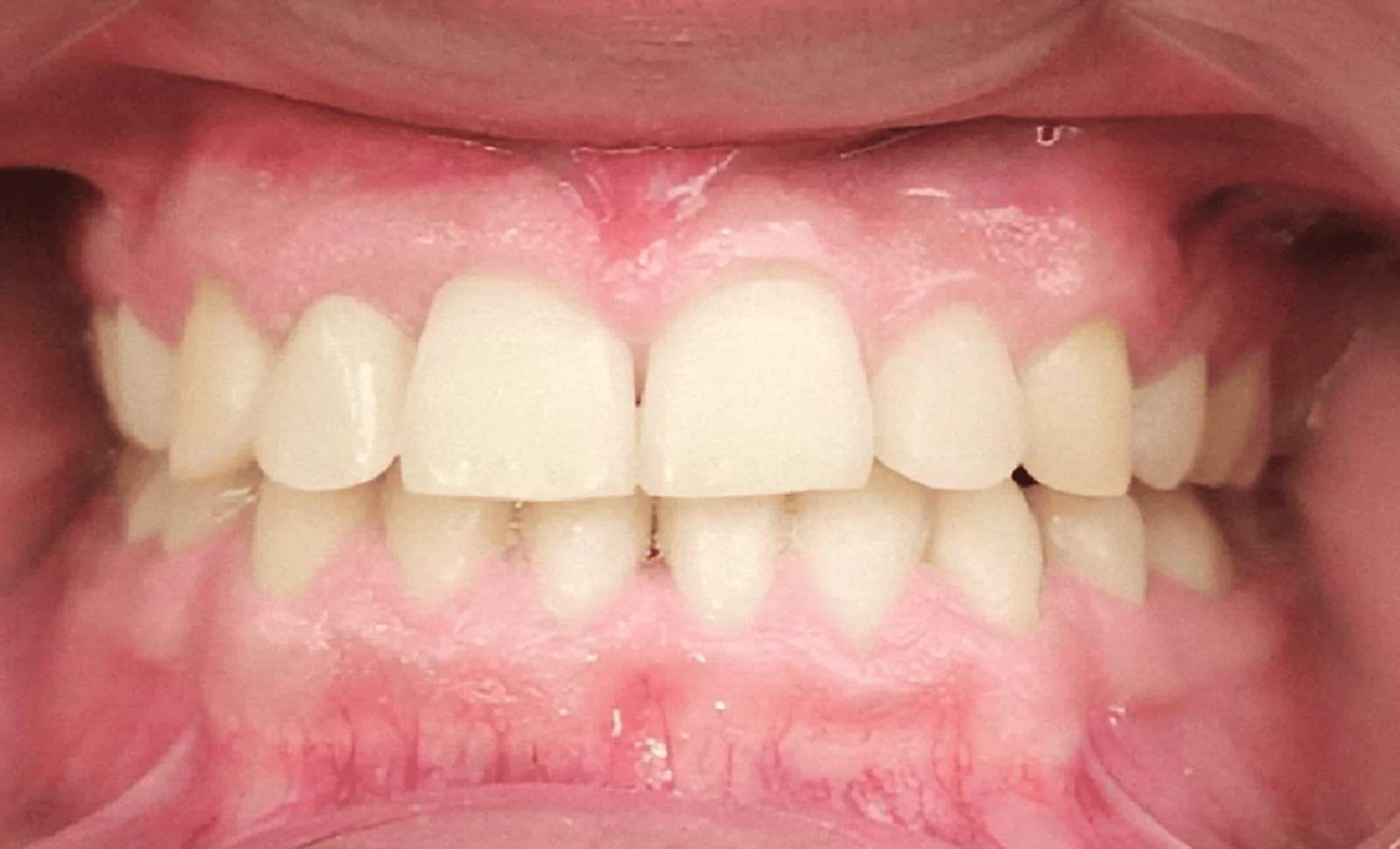
Final frontal intraoral photograph demonstrating the definitive implant-supported crown replacing the congenitally missing maxillary right lateral incisor (tooth #7) following a six-month osseointegration period. The definitive restoration exhibits excellent integration with the adjacent natural dentition, harmonious peri-implant soft tissue contours, proper tooth alignment, satisfactory occlusion, and favorable esthetic outcomes. Clinical evaluation confirmed successful functional and esthetic rehabilitation using a fully guided digital implant workflow.

After an appropriate osseointegration period, the implant was restored with a definitive implant-supported crown. Final clinical evaluation demonstrated excellent integration of the restoration with the adjacent natural dentition, harmonious gingival contours, proper tooth alignment, satisfactory occlusion, and favorable esthetic and functional outcomes. Postoperative clinical and radiographic assessment confirmed accurate implant positioning consistent with the original digital treatment plan (Figure 7). 

## Discussion

Computer-guided implant surgery has transformed implant dentistry by improving the precision and predictability of implant placement through the integration of CBCT, intraoral digital scanning, virtual implant planning, and CAD/CAM technology. Unlike conventional freehand implant placement, fully guided surgery enables prosthetically driven planning that considers both the restorative requirements and the available bone anatomy before surgery, thereby reducing the risk of implant malposition and improving restorative outcomes [1-4].

Congenital hypodontia of the maxillary lateral incisor is one of the most common developmental dental anomalies and frequently presents significant esthetic and functional challenges. Treatment options include orthodontic space closure, resin-bonded fixed partial dentures, conventional fixed dental prostheses, and implant-supported restorations. Following completion of craniofacial growth, implant-supported rehabilitation represents a conservative and predictable treatment modality because it preserves adjacent teeth, maintains alveolar bone, and provides excellent long-term esthetic and functional outcomes [5-8].

A major advantage of the digital workflow used in the present case was the integration of CBCT imaging with intraoral digital scanning. This approach allowed accurate 3D evaluation of the alveolar ridge, restorative space, implant angulation, emergence profile, and the relationship to adjacent teeth before surgery. Virtual planning also facilitated prosthetically driven implant positioning rather than relying solely on the available bone anatomy, thereby optimizing the restorative outcome while minimizing the risk of surgical complications [9-12].

The use of a CAD/CAM-generated tooth-supported surgical guide allowed the virtual treatment plan to be transferred accurately to the clinical setting. Guided implant surgery has been shown to reduce deviations in implant position, angulation, and depth compared with conventional freehand techniques, particularly in esthetically demanding regions such as the anterior maxilla. Accurate implant positioning is especially important when replacing maxillary lateral incisors because even minor deviations may compromise both esthetic appearance and prosthetic function [13-16].

The fully guided surgical approach also contributes to greater surgical efficiency and predictability. Precise osteotomy preparation minimizes unnecessary bone removal, facilitates implant placement according to the planned restorative position, and may reduce surgical time while increasing operator confidence. In addition, digital planning improves communication among the clinician, laboratory technician, and patient by allowing visualization of the anticipated treatment outcome before surgery [17-20].

In the present case, the digital workflow resulted in accurate implant positioning, excellent primary stability, uneventful healing, successful osseointegration after six months, and favorable esthetic and functional rehabilitation following placement of the definitive implant-supported crown. The harmonious peri-implant soft tissue contours and integration of the final restoration with the adjacent dentition demonstrated the clinical advantages of prosthetically driven implant planning in the esthetic zone.

Although the outcome of this case was favorable, several limitations should be considered. As a single case report, the findings cannot be generalized to all clinical situations. Furthermore, long-term follow-up is necessary to evaluate peri-implant tissue stability, prosthetic longevity, and maintenance over time. Future prospective clinical studies with larger patient populations are needed to further validate the long-term effectiveness and accuracy of fully guided implant surgery for the rehabilitation of congenital maxillary lateral incisor hypodontia.

Overall, this case demonstrates that the integration of CBCT imaging, intraoral digital scanning, virtual implant planning, and CAD/CAM surgical guide fabrication provides a predictable, minimally invasive, and prosthetically driven approach for implant rehabilitation. Careful digital treatment planning combined with fully guided implant placement can achieve excellent surgical accuracy, favorable esthetic integration, and successful functional rehabilitation in patients presenting with congenital absence of the maxillary lateral incisor.

## Conclusions

This case demonstrates that fully guided implant placement using CBCT, intraoral digital scanning, virtual implant planning, and CAD/CAM surgical guide fabrication provides a predictable and prosthetically driven approach for the rehabilitation of congenital hypodontia of the maxillary right lateral incisor. The digital workflow facilitated accurate implant positioning, successful osseointegration, favorable peri-implant soft tissue healing, and excellent esthetic and functional outcomes following definitive implant-supported restoration. Although additional long-term studies are needed to further validate this approach, the present case highlights the clinical value of integrating digital technologies to improve surgical precision, treatment safety, and restorative predictability in implant rehabilitation.
